# Increasing quality stability of jasmine tea beverage by encapsulation from protein-polysaccharide nanocomplexes

**DOI:** 10.1038/s41538-025-00703-5

**Published:** 2026-01-08

**Authors:** Hujun Xie, Han Wang, Min Huang, Zhenbang Zhou, Ying Gao, Qing-Qing Cao, Qingbo Jiao, Gerui Ren, Yong-Quan Xu

**Affiliations:** 1 School of Food Science and Biotechnology, Zhejiang Gongshang University, Hangzhou, China; 2 Key Laboratory for Food Microbial Technology of Zhejiang Province, School of Food Science and Biotechnology, Zhejiang Gongshang University, Hangzhou, China; 3 Tea Research Institute Chinese Academy of Agricultural Sciences, State Key Laboratory for Tea Plant Germplasm Innovation and Resource Utilization, Key Laboratory of Biology, Genetics and Breeding of Special Economic Animals and Plants, Ministry of Agriculture and Rural Affairs, Hangzhou, China

**Keywords:** Biochemistry, Biological techniques, Nanoscience and technology

## Abstract

As widely consumed beverage, the stability is crucial to the quality and shelf life of tea beverage. In this study, whey protein isolate (WPI)-beta-cyclodextrin (β-CD) was added to investigate its effect on the stability of jasmine tea beverage by pH, thermal and storage stability. The results showed tea beverages under neutral and acidic conditions are more stable than those under alkaline conditions, and have higher antioxidant activity and total phenol retention rate. The storage stability experiments (25 °C for 30 d, and 4 °C for 150 d) showed the higher the storage temperature, the less stable the tea beverages were. WPI-β-CD addition was beneficial to delaying the degradation of tea polyphenols in the tea beverages, delayed by 6.41% after 150 d of storage. This study provides a theoretical basis for the development of new tea beverage products and the wide application of EGCG.

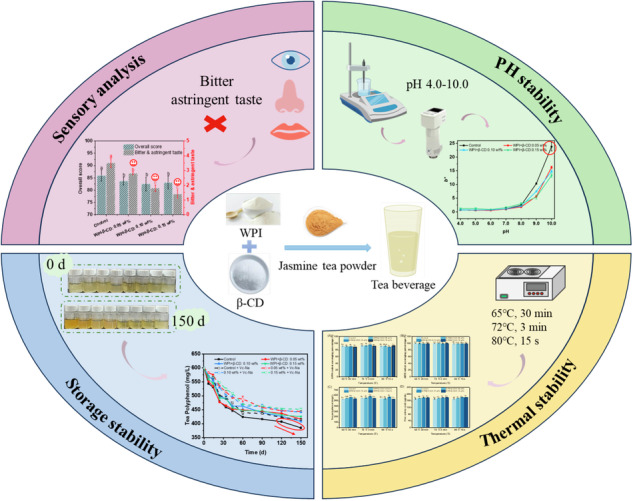

## Introduction

Jasmine tea is one kind of scented tea that is reprocessed from processed green, oolong, black, and other tea by absorbing the fragrance of fresh flowers^1^. In recent years, jasmine tea became increasingly popular due to its unique flavour and various health benefits, including effects on hypoglycaemic activities, regulating immunity and antioxidant properties^2^. According to statistics, the global tea beverage market is growing at a steady rate, especially among young consumers, showing strong consumption potential. Although tea beverage has advantages in taste and bioactivity, it faces multiple challenges in stability during production and storage^3^. These challenges mainly stem from the active ingredients in tea extracts, such as tea polyphenols and free amino acids^4^. These ingredients are easily affected by environmental factors, such as light, heat, oxygen, and microorganisms, resulting in a decline or even deterioration in the quality of tea beverage. Sang, Lee, Hou, Ho, & Yang^5^ found that higher oxygen content and lower concentrations of antioxidants increased the oxidation of catechins. Wang, Zhou, & Wen^6^ found that heating led to the epimerization of tea catechins. Han et al.^7^ indicated that pure tea beverage was susceptible to temperature, and after heat treatment, their antioxidant capacity was gradually reduced. Therefore, the stability of tea beverage is crucial to its quality and shelf life. The instability of tea beverage results in colour change, precipitation, taste deterioration, and other phenomena, thus affecting the sensory experience of consumers.

Nanotechnology has multiple applications in the food sector, including food safety and quality, food packaging and sensory improvement, targeted delivery of compounds, and increased bioavailability^8,9^. Phenolic substances, including tea polyphenols, play an active role in food processing and packaging. The nano-encapsulation systems can guarantee the colour and quality of polyphenols and improve the performance of packaging materials^10,11^. It was reported that resveratrol and (-)-Epigallocatechin-3-gallate (EGCG) were encapsulated by low-methylated pectin-coated nano-liposomes, and then added to orange juice. The nano-liposomes maintained good stability and had stronger antioxidant activity after sterilisation^12^.

EGCG is one of the main components responsible for the bitterness and astringency of tea. A sodium caseinate-EGCG complex was successfully prepared using zein nanoparticles as emulsifiers and sodium caseinate and EGCG as raw materials. This complex can effectively reduce the chance of EGCG coming into contact with oral receptor cells, thereby achieving the purpose of eliminating the bitter taste of EGCG^13^. Whey protein isolate (WPI) is mainly composed of β-lactoglobulin (~65%) and α-lactalbumin (~25%), and is recognised as one of the high-quality protein supplements for the human body. Meanwhile, WPI is a by-product of cheese production. Under the current production and consumption patterns, it is seriously wasted and has a relatively low price^14^. For achieving efficient utilisation of WPI, it was used to form complexes with pectin to enhance the stability of anthocyanin-based colour in a model beverage system^15^. β-cyclodextrin (β-CD) is a product formed by the hydrolysis and cyclisation of starch, featuring a hydrophobic central cavity and a hydrophilic outer surface. Its unique structure enables it to act as a host molecule and form complexes with various guest molecules that have hydrophobic groups^16^. It has been reported that encapsulating naringin with β-CD can reduce the bitterness of grapefruit juice while maintaining its original flavour and nutritional value^17^.

In this study, nano-encapsulation was applied to tea beverage. Whey protein isolate (WPI)-beta-cyclodextrin (β-CD) was added to jasmine tea powder to prepare jasmine tea beverage. Sensory evaluation was carried out to assess whether WPI-β-CD nanocomplexes were able to mask the bitterness and astringency. The stability of tea beverage was analysed from three aspects: pH stability, thermal stability and storage stability. The antioxidant activity, total phenolic content, free amino acid content, turbidity, and colour difference of tea beverage samples under different conditions were measured to assess the stability. The results provide a theoretical basis for developing a new tea beverage with high quality and stability.

## Results and discussion

### Sensory evaluation

**Fig. 1:**
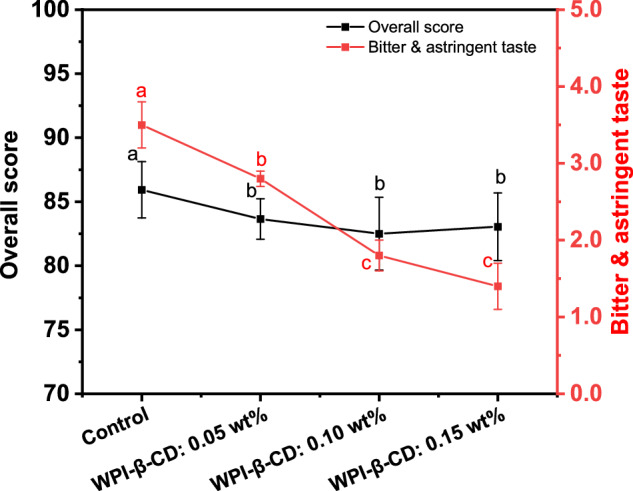
Sensory evaluation of tea beverages treated with different concentrations of WPI-β-CD. Different letters mean significant differences (*p* < 0.05).

According to the sensory analysis of tea beverages under different treatment conditions, it can be seen from Fig. 1 that the overall score of the Control group was higher than that of the WPI-β-CD treatment groups, probably because the addition of WPI increased the turbidity of tea beverages. It is noteworthy that the addition of WPI-β-CD increased the aroma of tea beverages, making the decrease of the overall score less than the decrease of the soup colour score between the WPI-β-CD treatment and the Control groups. This might be due to the unique molecular structure of β-CD, which can form inclusion complexes with aroma molecules. Encapsulating the aroma molecules makes them more stable and slows down their volatilisation rate^18^. As for the bitterness and astringency of tea beverages, the Control group was the highest. And the bitterness and astringency gradually decreased with the increase of WPI-β-CD addition. It might be attributed to the hydrophobic cavity of β-CD, which allowed the galloyl groups that caused the bitterness and astringency of EGCG to enter the interior of the hydrophobic cavity and produce hydrogen bond interactions, thus preventing the binding to the bitter taste receptor and reducing the bitterness and astringency of EGCG^19^. More WPI-β-CD indicated that more bitter and astringent substances in the tea beverages were bound, masking the bitterness and astringency of the tea beverages^20^.

### pH stability analysis

**Fig. 2:**
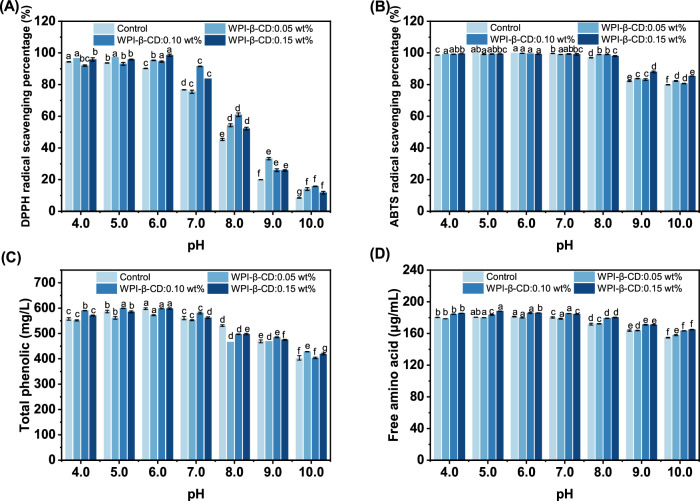
Stability evaluation parameters of tea beverages at different pH values. **A** Antioxidant activity of DPPH; **B** antioxidant activity of ABTS; **C** total phenolic concentration; **D** free amino acid concentration. Different letters mean significant differences (*p* < 0.05).

Analysing the stability of tea beverages under different pH values, understanding the influence of pH on the chemical reactions, colour, flavour, nutritional components and microbial growth of tea beverages, is conducive to optimising storage conditions, extending the shelf life and maintaining product quality^21^. The antioxidant activities of tea beverages at different pH values are shown in Fig. 2A, B. When pH ≤ 6.0, the DPPH free radical scavenging ability of the Control group was maintained at 90.23–97.27%. When pH > 6.0, it gradually decreased and dropped to the lowest (8.44%) at pH 10.0. The change trend of WPI-β-CD treatment groups was consistent with that of Control group, but the decrease was significantly lower than that of Control group, which indicated that WPI-β-CD had a protective effect on the active substances in tea beverages. When pH ≤ 8.0, the ability of scavenging ABTS free radicals in the Control group was stronger, which was maintained at 96.92–100%. When pH > 8.0, it decreased significantly and reached the lowest level of 79.79% at pH 10.0. The antioxidant activity of tea beverages in acidic and neutral environments was stronger than that in alkaline conditions. It might be attributed to that the active substances in tea were unstable under alkaline conditions, which resulted in a decrease in antioxidant activity^22^. Another possible reason was that changes in hydrogen ion concentration led to changes in the mechanism of scavenging DPPH and ABTS free radicals by phenolic compounds^23^.

The concentration of total phenolics in the initial tea beverages was 600 mg/L. The concentration of total phenolics in tea beverages adjusted to different pH values is shown in Fig. 2C. The loss rate of total phenolics increased with the increase of pH value of the beverages, so it was believed that low pHs had a protective effect on total phenolics of tea beverages. At pH 4.0, the total phenolics content of tea was reduced. It was reported that the caffeine-catechin interaction could lead to precipitation at low pH^24^. The catechins involved in the insoluble caffeine-catechin complex were calculated as catechin loss due to the physical instability of catechins^25^. From this point of view, the total phenolic content of tea was also reduced. At pH 5.0 and 6.0, the change of total phenolic concentration was not obvious, which indicated that tea polyphenols had strong stability under weak acidic and neutral conditions. When pH > 6.0, total phenolic concentration decreased significantly. Previous studies revealed that tea polyphenols were oxidised into quinones mainly through oxidation and dimer formation, which led to the decrease of total phenolic content^26,27^. Our results suggested that tea polyphenols were relatively stable under acidic conditions, which were in accordance with the findings of Roginsky & Alegria^28^. Ananingsih et al.^29^ also drew a similar conclusion.

It can be seen from Fig. 2 that there was a close correlation between the DPPH scavenging ability and total phenolics, especially in the late period when the DPPH scavenging ability of tea beverages decreased significantly. Under strong alkaline conditions, the chemical structure of phenolics was significantly changed. The phenolic hydroxyl group (-OH) in phenolics was deprotonated (dehydrogenated or changed to a phenolate form)^30^, resulting in a decrease in their antioxidant capacity without a significant decrease in total phenolic content. In addition, the antioxidant components in tea beverages not only included phenolics, but also some other substances, such as amino acids, vitamin C, minerals, and so on^31^. Changes in these components under strong alkaline conditions might affect the overall antioxidant activity.

Free amino acids are important components in tea. Studies have shown that high levels of free amino acids are beneficial to the quality of green tea^32^. The concentration of free amino acids in tea beverages at different pHs is shown in Fig. 2D. As the addition of protein was low, the effect of WPI on free amino acid content was negligible. In the Control group, the free amino acid concentration was 180.29 μg/mL at pH 4.0, increased to 181.63 at pH 6.0, and decreased to 154.70 μg/mL at pH 10.0 μg/mL. The free amino acid concentration showed a trend of first increasing and then decreasing. This indicated that tea beverage can maintain a higher free amino acid concentration under neutral conditions. The groups treatment with WPI-β-CD also showed the same trend, but the degree of free amino acid reduction was lower than that in the Control group. This indicated that WPI-β-CD had a protective effect on free amino acids in tea beverages at different pH. Liu et al.^33^ studied the effects of different pH water quality on the sensory quality of roasted green tea, and concluded that the pH value of water had no significant role in the influence of free amino acid leaching.

**Fig. 3:**
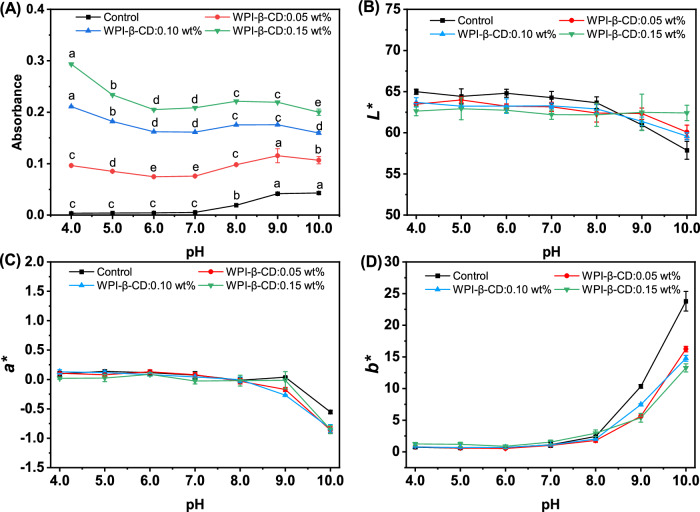
Stability evaluation parameters of tea beverages at different pH values. **A** Turbidity; **B** L* of colour; **C** a* of colour; **D** b* of colour. Different letters mean significant differences (*p* < 0.05).

The turbidity of the tea beverages at different pH levels is shown in Fig. 3A. The addition of WPI increased the turbidity of the beverage system. The turbidity of the tea beverage was larger at pH 4.0–5.0. It was due to the fact that the isoelectric point of WPI was between pH 4.0–5.0. When the pH value was close to the isoelectric point of WPI, it led to the aggregation of WPI and an increase in turbidity. Under alkaline conditions, the proteins in tea (such as tea proteins) may undergo structural changes, resulting in protein aggregation or precipitation. Proteins combine with polyphenolic substances to form complex polymers or particles, causing an increase in the turbidity of the tea beverage^34^.

The influence of pH on the chroma of tea beverages is shown in Fig. 3B–D. When pH was 4.0–8.0, L* was in the range of 64.99–62.39 and the overall fluctuation was not large, indicating that the influence on L* was not large in the pH range. When pH > 8.0, L* dropped significantly, which was related to the oxidative deterioration of tea polyphenols in tea beverages^27^. a* indicates the degree of red. The larger the a* value, the redder the colour is. b* indicates the degree of yellow. The larger the b* value, the yellower the colour is. When pHs ranged between 4.0–8.0, a* and b* changed little. When pH > 8.0, tea beverages became yellower, with a* decreased slightly and b* increased greatly. Under alkaline conditions, tea polyphenols could be easily oxidised to quinones and thearubigins were prone to converting into theabrownins, both of which led to decreased a*, less redness, more yellowness, and even brown^30,35^. Moreover, b* in the Control group was significantly increased compared to the WPI-β-CD treatment groups, indicating that our treatment had a positive effect on the colour preservation of tea beverages.

### Thermal stability

**Fig. 4:**
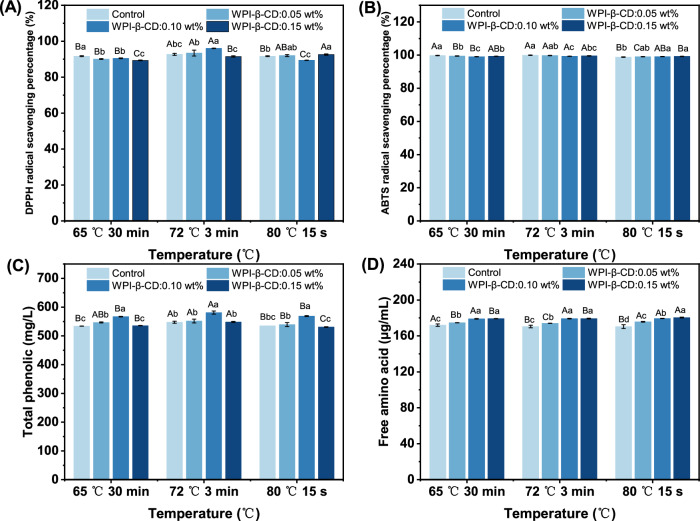
Stability evaluation parameters of tea beverage under different heat treatment conditions. **A** Antioxidant activity of DPPH; **B** Antioxidant activity of ABTS; **C** total phenolic concentration; **D** free amino acid concentration. Different uppercase letters indicate significant differences (*p* < 0.05) between the same samples at different temperatures, and different lowercase letters in the graph indicate significant differences (*p* < 0.05) between different samples at the same temperature.

According to the method of Karakaş, Yildirim, and Karadag^36^, pasteurisation temperatures were selected as 65 °C for 30 min, 72 °C for 3 min, and 80 °C for 15 s, respectively, to analyse the thermal stability of tea beverage. DPPH free radicals have unpaired electrons, look purple, and have a maximum absorption wavelength of 517 nm. By receiving hydrogen atoms or electrons, the DPPH radical turned into stable DPPH-H, which weakened its absorption capacity and appeared a lighter colour^37^. ABTS free radicals were blue, with a maximum absorption wavelength of 734 nm. Antioxidant compounds reacted with ABTS free radicals, weakened their absorption capacity, and made the solution lighter in colour^38^. The antioxidant activity of tea beverages under different heat treatment conditions is shown in Fig. 4A, B. Temperature had an influence on the scavenging of DPPH free radicals in tea beverages, because higher temperature led to the degradation of phenolics and significantly decreased the scavenging rate of DPPH free radicals^39^. In addition, heating had an effect on the epimerization of catechins. EGCG, EGC, EC, and ECG underwent epimerization during heating, and therefore, the antioxidant activity decreased after heating^40^. However, temperature had little effect on scavenging ABTS free radicals. It is that the difference of the substituent group on the D ring of EGCG may cause the different electron-transfer reactions and H atom transfer capabilities, which could lead to different results measured by the ABTS and DPPH assays^41^.

Heat treatment was important in the manufacture of canned and bottled tea beverages to extend their shelf life^29^. The concentration of total phenolics under different heat treatment conditions is shown in Fig. 4C. Under the treatment condition of 65 °C, the total phenol content of all tea beverages was relatively low, especially the concentration of the Control group was 533.83 mg/L. Under the treatment condition of 72 °C, the total phenol content generally increased, especially in the WPI-β-CD:0.10 wt% group, with a concentration of 581.18 mg/L. At 80 °C treatments, the total phenol content slightly decreased. The Control group was more significantly (534.97 mg/L). This might be because the long heating time at a lower temperature (65 °C) increased the oxidation and degradation time of phenolic substances in the tea beverage, resulting in a decrease in the total phenol content. However, high-temperature conditions (80 °C) accelerated the degradation of phenolic substances and also reduced the total phenolic content. With the increase in temperature, the oxidative decomposition rate of phenolics became faster. The tea beverages added with WPI-β-CD clearly showed less reduction of total phenolic concentration than that in the Control group. In other words, the addition of WPI-β-CD improved the thermal stability of phenolics and slowed down their degradation. This phenomenon can be attributed to the interactions formed between WPI-β-CD and phenolics, which has been proved in our previous research^19^. These interactions made the complex structure be more stable, providing a shielding and protective effect against heat-induced degradation. Zeng et al.^27^ found that total catechins decreased with increasing temperature in the prepared tea polyphenol solution, indicating that some catechins were oxidised.

Free amino acids are the main flavour substances in the freshness of tea soup. The concentration of free amino acids in tea beverage after heat treatment at different temperatures is shown in Fig. 4D. The change of free amino acid concentration in tea beverage was consistent with that of total phenolic concentration. The free amino acid content of all tea beverages at 65 °C was generally high, especially in the WPI-β-CD:0.15 wt% group (179.23 μg/mL). As the temperature increased and the processing time shortened, the content of free amino acids slightly decreased, which may be related to the degradation of heat-sensitive components. High temperature reduced the content of free amino acids, and the addition of WPI-β-CD could reduce the loss of free amino acids in the tea beverage system, which meant that WPI-β-CD had a protective effect on the ingredients in the tea beverage.

**Fig. 5:**
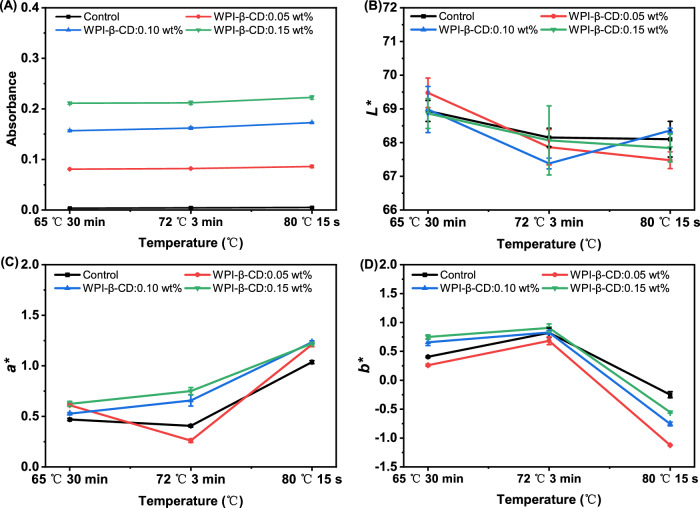
Stability evaluation parameters of tea beverage under different heat treatment conditions. **A** Turbidity; **B** L* of colour; **C** a* of colour; **D** b* of colour.

The turbidity of tea beverage under different heat treatment conditions is shown in Fig. 5A. Temperature had little effect on the turbidity of tea beverage. The turbidity increased slightly at 80 °C for 15 s, probably because high temperature led to protein denaturation and increased protein condensation and precipitation^42^.

The colour difference of tea beverage under different heat treatment conditions is shown in Fig. 5. With the increase of temperature, L* of tea beverage slightly decreased, and the decrease of L* was related to the heating temperature and time. As the heating temperature increased, L* decreased. On the contrary, a* rose with the increase of heating temperature. These findings suggested that the overall colour of the tea beverages diminished post-treatment, with the infusion’s brightness gradually declining and shifting towards reddish hues^43^. The pronounced alteration in colour of the tea infusion was primarily attributed to the oxidation and degradation of certain phenolic compounds, and the formation of the Maillard reaction products^44^. For instance, oxidative degradation products, including (2R)-2-Hydroxy-3-(2,4,6-trihydroxyphenyl)-1-(3,4,5-trihydroxyphenyl)-1-propanone 2-*O*-gallate, theacitrin C/dehydrotheasinensin AQ theacitrinin A, EGCG Quinone Dimer B and theasinensin A, may contribute to this phenomenon^45^. Previous research also indicated that heat treatment and prolonged storage significantly impacted the changes in flavour metabolites and sensory quality of tea beverages^46,47^. b* showed a downward trend at 80 °C for 15 s, which might be related to the shorter heating time.

### Storage stability

**Fig. 6:**
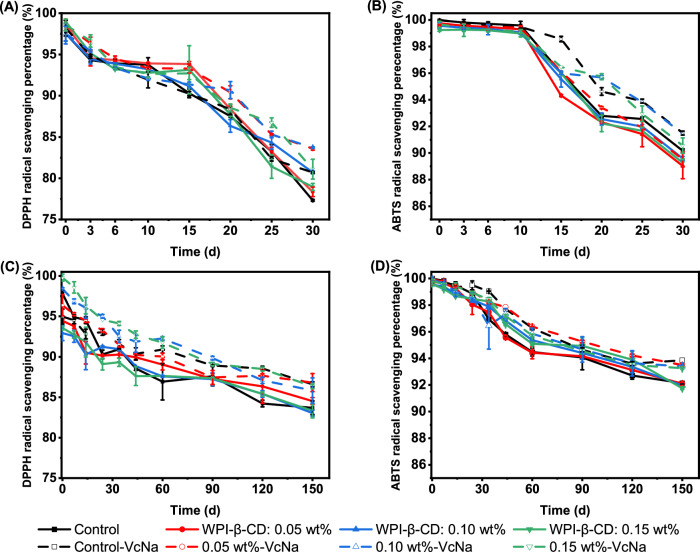
Stability evaluation parameters of tea beverage under different storage conditions. **A**, **B** Antioxidant activity of DPPH and ABTS at 25 °C. **C**, **D** Antioxidant activity of DPPH and ABTS at 4 °C.

During the storage process of tea beverages, due to the influence of external conditions (such as temperature), the DPPH free radical scavenging ability and ABTS free radical scavenging ability, which reflected the antioxidant capacity of tea beverages, changed. As can be seen from Fig. 6, with the extension of storage time, both DPPH and ABTS scavenging capacity of tea beverages showed a downward trend. At 25 °C, the DPPH scavenging capacity and ABTS scavenging capacity of tea beverage in the Control group decreased by 19.75% and 8.65%, respectively. Under 4 °C storage conditions, DPPH scavenging capacity decreased by 14.09% and ABTS scavenging capacity decreased by 8.78%. The increase in temperature accelerated the degradation of antioxidant substances in tea beverages, resulting in the decrease of DPPH and ABTS scavenging capacity^48^. In addition, Alvarado-López, Parralejo-Sanz, Lobo, & Cano^49^ found that the DPPH clearance of Brazil nut beverages with *Opuntia stricta* var. *dilleniid* fruit pulp extracts decreased as the total phenols decreased during storage. Moreover, high storage temperature tended to cause a more obvious decrease in DPPH clearance ability. Kim et al.^50^ also found that the antioxidant activity of kiwi puree was decreased with higher storage temperature and longer storage time. However, the addition of V_C_Na played an important role in slowing down the decrease of DPPH and ABTS scavenging ability^51^. At 25 °C, the DPPH scavenging ability of tea beverage in Control-V_C_Na group decreased by 17.93% and the ABTS scavenging ability decreased by 7.45%. Under 4 °C storage conditions, DPPH scavenging capacity decreased by 10.88% and ABTS scavenging capacity decreased by 6.14%, demonstrating that V_C_Na acted as an antioxidant and played a crucial role in preserving the antioxidant activity of tea beverages^52^. In addition, the free radical scavenging ability of the treated tea beverage after adding WPI-β-CD was higher than that of the Control group, indicating that nano-encapsulation was helpful to protect the active substances in the tea beverage and improve the antioxidant activity of the tea beverage.

During the storage of tea beverage, the retention of total phenolics was an important index reflecting the quality change of tea beverage^53^. As can be seen from Fig. S1, with the extension of storage time, total phenolics in tea beverages showed a decreasing trend. At 25 °C, the content of total phenolics in tea beverage changed significantly. After 30 days of storage, the content of total phenolics in the Control group decreased by 51.51%, and the total phenolic content in the WPI-β-CD treatment groups decreased by 42.50%, 40.13% and 42.50%, respectively. Under the condition of 4 °C, the content of total phenolics in the Control group decreased by 35.58% after 150 days of storage. The total phenolic content in the WPI-β-CD treatment groups decreased by 30.80%, 32.50%, and 29.17%, respectively. It indicated that the degradation of phenolics in tea beverages was accelerated by the increase in temperature. WPI-β-CD had a protective effect on phenolics and slowed down the decrease of total phenolic content. Oxidation and dimerisation of phenolics occurred during storage, which caused the content of phenolics to decrease gradually. During storage, catechins oxidised and dehydrogenated to form quinones, and then polymerised to form theabrownins. Catechins and their oxidation products also reacted with amino acids and proteins^54^. Similarly, Ma et al.^55^ found that under the storage conditions at 25 °C, the changes in turbidity and catechin content were significantly greater than those at 4 °C, indicating that the increase in temperature accelerated the degradation of phenolics. For Control-V_C_Na stored at 25 °C for 30 days, the content of total phenolics decreased by 48.90%, and that at 4 °C for 150 days decreased by 30.93%, indicating that the addition of V_C_Na had a better protective effect on the content of total phenolics in tea beverages. It was also found that the content of total phenolics in all tea beverages added with WPI-β-CD was higher than that in the Control group, indicating that WPI-β-CD had a protective effect on phenolics during long-term storage, which was consistent with our previous findings^19^.

Figure S1 showed the concentration of free amino acids in tea beverages at different storage temperatures. In the Control group, the initial free amino acid concentration was 181.24 μg/mL, which decreased by 20.4% after 30 days of storage at 25 °C and 15.78% after 150 days of storage at 4 °C. With the increase of storage time, the content of free amino acids decreased, and the higher the storage temperature, the more obvious the decrease of free amino acid content was. High temperature caused amino acids to degrade through various chemical reactions, including the Maillard reaction and oxidation^56^. After adding WPI-β-CD, the decrease of free amino acid content in tea beverage was lower than that in the Control group, and the decrease of free amino acid content in tea beverage after adding VcNa was significantly lower than that in the control group, indicating that both WPI-β-CD and VcNa had protective effects on free amino acids. Chen, Zhu, Tsang, & Huang^57^ suggested that additives used in beverage production, such as citric acid or ascorbic acid, might interact with green tea catechins and affect their stability.

Tea cheese refers to an obvious precipitation phenomenon, which is a mixture of various biochemical components in tea beverage through hydrogen bonds and hydrophobic interaction. As shown in Fig. S1, the turbidity of tea beverages increased at both 4 °C and 25 °C. The turbidity rose faster in the early stage of storage, indicating that the early stage of storage was the main stage of precipitation formation. Zhou et al.^58^ also found the same phenomenon during the storage of tea brewing liquid.

As shown in Fig. S2, The L* of tea beverage gradually decreased during storage, and the change trend was consistent under different storage conditions. When stored at 25 °C, L* of tea beverage in the Control group decreased by 13.24% at 0–6 days, which was at a fast decrease rate, and by 3.50% at 6–30 days, which was at a relatively slow decrease rate. When stored at 4 °C, L* of tea beverage in the Control group decreased by 9.12% at 0–14 days and 14.81% at 14–150 days. The oxidation and degradation of polyphenols and chlorophyll substances in tea beverages under the influence of high temperature, light, oxygen, and other factors during storage, resulting in the deepening of the colour of tea beverages^59^. Therefore, L* of tea beverages gradually decreased during storage. This is consistent with the results of Chen et al.^60^. L* in the group with VcNa was slightly higher than that in the group without VcNa, suggesting the protective effect of V_C_Na on L* in tea beverages during storage^61^. L* in tea beverages treated with WPI-β-CD was slightly lower than that in the Control group, because the addition of protein caused the brightness of the tea beverage to decrease.

Under the conditions of 25 °C and 4 °C, a* increased gradually with the extension of storage time, and the increase rate was greater with the increase of storage temperature. When the Control group was stored at 25 °C for 30 days, a* increased from −1.11 to 0.93. After being stored at 4 °C for 150 days, a* increased from −1.11 to 0.83, which might be attributed to the oxidative degradation of phenolics and chlorophyll during the storage process of tea beverages, causing the tea beverages to lose their original yellow-green brightness and become red, dark, or even brown^59^.

The b* increased gradually with the increase of storage time. In the Control group, when stored at 25 °C for 30 days, b* increased from −1.56 to 3.93. When stored at 4 °C for 150 days, b* increased from −1.56 to 4.28. The increase of b* was related to the browning of tea beverages caused by the oxidation of phenolics. In addition, it was obvious that the increase of b* in the VcNa-added group was smaller than that in the VcNa-absent group, which indicated that VcNa had a significant protective effect on the colour of tea beverages. The increase of b* in the WPI-β-CD treatment groups was smaller than that in the Control group. It was speculated that the interactions between WPI-β-CD and the phenolic substances in the tea beverage could slow down the browning process of the tea beverage, which further indicated that our treatment was helpful to improve the stability of tea beverages. According to the change trend of L*, a*, and b*, it was found that all the tea beverage samples showed a decrease in brightness and an increase in redness and yellowness during storage. In terms of sensory aspects, the brightness of tea beverages decreased, and the colour turned red and brown^62^. Moreover, the higher the storage temperature, the more obvious the colour change trend of tea beverages became. Chen et al.^60^ studied the quality changes of different tea beverages during storage, and found that L* of each tea beverage gradually decreased and a* and b* gradually increased with the extension of storage time, which was consistent with the results of this experiment.

In this study, WPI-β-CD was applied to tea beverage, and the sensory experiment showed that the tea beverage after adding WPI-β-CD could mask the bitterness and astringency of the original tea beverage. The stability of tea beverage was analysed comprehensively from three aspects: pH stability, thermal stability, and storage stability. Under different pH conditions, phenolics underwent oxidation or polymerisation reactions, and the content of free amino acids also changed. The results of the pH stability experiment showed that tea beverages were more stable under acidic or neutral conditions. The thermal stability test indicated that phenolics were easily degraded at high temperature. In addition, tea beverages were easily affected by temperature during storage, which led to deterioration. After 30 d of storage at 25 °C, the content of total phenolics in the Control group was significantly lower than that in the WPI-β-CD treatment groups. The same phenomenon was also observed in samples after 150 days of storage at 4 °C. VcNa had an obvious protective effect on the flavour quality and biochemical components of the tea beverage during sterile storage. The results of the storage experiment showed that the storage condition of 4 °C was more conducive to the stability of the tea beverage, and the addition of VcNa had a protective effect on the active ingredients in the tea beverage. It provides a theoretical basis for the production and storage of tea beverages. In future, the content changes of each catechin monomer can be measured to gain a deeper understanding of the impact of each content change on the functional characteristics of tea beverages.

## Methods

### Materials and reagents

Food-grade β-CD (≥98%) was purchased from Henan Wanbang Chemical Technology Co., LTD. (Henan, China). Food-grade WPI (≥80%) was purchased from Shanghai Wankang Biotechnology Co., LTD. (Shanghai, China). Jasmine tea powder was gifted from the Tea Research Institute, Chinese Academy of Agricultural Sciences (Hangzhou, China). Food-grade L-ascorbic acid sodium salt (VcNa) was obtained from Zhongchen Biotechnology Co., LTD. (Zhengzhou, Henan). Ninhydrin, SnCl_2_, Na_2_HPO_4_, and KH_2_PO_4_ were purchased from Shanghai Aladdin Reagent Co., LTD. (Shanghai, China). Potassium tartrate, 2,2-diphenyl-1-picrylhydrazyl (DPPH), 2,2-azino-bis (3-ethylbenzothiazoline-6-sulfonic acid) (ABTS) and Fe_2_SO_4_ were purchased from McLean Biochemical Technology Co., LTD. (Shanghai, China). Pure water used in this experiment was purchased from Hangzhou Wahaha Group Co., Ltd. (Hangzhou, China).

### Preparation of jasmine tea beverage

Previous study by Xie et al.^19^ showed that the optimal ratio of WPI-β-CD encapsulated EGCG was 1:3. Thus, the ratio of WPI/β-CD was kept at the optimal ratio (WPI: β-CD = 1:3). The concentrations of WPI-β-CD powder were set at 0.05 wt%, 0.10 wt%, and 0.15 wt%. WPI-β-CD complex was weighed and dissolved in water and stirred evenly with a magnetic stirrer at room temperature. In this study, tea powder instead of EGCG was used as the core material. The content of total phenolic of tea powder was determined in advance by the iron (II) D-tartrate colourimetric method. Tea powder was added to the WPI-β-CD solution to reach a final concentration of total phenolic of 600 mg/L. The samples were recorded as Control, WPI-β-CD: 0.05 wt%, WPI-β-CD: 0.10 wt%, and WPI-β-CD: 0.15 wt%, respectively.

### Sensory experiment

Ten food professionals (male:female = 1:1, 20–25 years old) were randomly selected for sensory evaluation, referred to GB/T 31740.1-2015 “Tea products Part 1: Solid Instant Tea” and evaluated the food by colour, odour, and taste. In this study, the method was improved, and the weights of evaluation factors were set as: soup colour 20%, aroma 40%, and taste 40%. Using the percentage system, the summed scores' average was taken as the overall score. Bitterness and astringency were divided into 4–5, 3–4, 2–3, 1–2 and 0–1 regions, corresponding to “extremely strong”, “strong”, “neutral”, “weak”, and “extremely weak”, respectively^63^.

### Preparations of tea beverage samples for the stability study

The pH stability was carried out. According to the method of Liu, Sun, Xue, and Gao^21^, the pH value of tea beverage was adjusted to 4.0–10.0 with 0.1 M NaOH or 0.1 M HCl, and the stability analysis was carried out.

The thermal stability was carried. The pH of the initial tea beverage was adjusted to 6.0. According to the method of Raikos^64^ with slight modifications, the pasteurisation temperatures for thermal stability analysis were 65 °C for 30 min, 72 °C for 1 min, and 80 °C for 10–15 s, respectively.

The storage stability was carried. According to the method of Xu et al.^65^, 0.05% V_C_Na was added to each bottle of tea beverage by volume, which were recorded as Control-V_C_Na, 0.05 wt%-V_C_Na, 0.10 wt%-V_C_Na, and 0.15 wt%-V_C_Na, respectively. The pH of the fixed tea beverage was 6.0. Canning tea beverages were prepared using PET bottles. After boiling and sterilising, half of the canned tea beverage in each group was stored in the refrigerator at 4 °C for 150 days, and the other half was stored in the incubator at 25 °C for 30 d. The storage stability of tea beverage was analysed.

### Parameters for assessing the stability of tea beverage

According to the method of Yi, Lam, Yokoyama, Cheng, & Zhong^66^, a certain amount of DPPH powder was weighed and dissolved in anhydrous ethanol (0.1 mM DPPH solution) and stored for 30 min away from light. The sample solution (5 mL) was thoroughly mixed with 5 mL of DPPH solution and labelled as sample group A_1_. The 5 mL sample was thoroughly mixed with 5 mL of anhydrous ethanol and labelled as blank group A_2_. The group of 5 mL DPPH solution and 5 mL distilled water was labelled as control group A_0_, and all samples were stored at room temperature for 30 min away from light. The absorbance of sample group A_1_, blank group A_2_ and control group A_0_ was measured by an ultraviolet spectrophotometer at 517 nm. The DPPH free radical scavenging activity of the sample was calculated according to Eq. (1).

1 $$DPPH\,radical\,scavenging\,activity( \% )=\left(1-\frac{{A}_{1}-{A}_{2}}{{A}_{0}}\right)\times 100$$

The pre-prepared ABTS mixture stocking solution (7.4 mM ABTS + 2.6 mM K_2_S_2_O_8_) was stored overnight. Before use, the ABTS mixture stocking solution was diluted with distilled water until its absorbance at 734 nm was adjusted to 0.70 ± 0.05. The diluted ABTS mixture solution (6 mL) was mixed with 2 mL of the sample solution. The absorbance of the ABTS mixture solution after adding the sample was A_a_, the absorbance of distilled water with sample was A_b_, and the absorbance of ABTS mixed solution with distilled water was A_c_. All samples were kept in the dark at room temperature for 20 min, and their absorbance values were measured at 734 nm. The ABTS free radical scavenging activity of the sample was calculated according to Eq. (2).

2 $$ABTS\,radical\,scavenging\,activity( \% )=\left(1-\frac{{A}_{a}-{A}_{b}}{{A}_{c}}\right)\times 100$$

According to the method of Liang, Lu, Zhang, Wu, & Wu^67^, 25 mL of the sample was added into a 50 mL volumetric bottle, mixed with 15 mL of 95% ethanol, placed for 15 min, and then water was added to reach the total volume of 50 mL. The solution was filtered with slow-speed quantitative filter paper, and the filtrate was set aside. 5 mL of the filtered tea beverage sample was put into a 50 mL graduated colourimetric tube, 4 mL of water and 5 mL of ferrous tartrate solution were added, and the mixture was thoroughly mixed. Phosphate buffer (pH = 7.5) was added to reach a total volume of 25 mL, and the absorbance of the solution was determined at 540 nm using an ultraviolet spectrophotometer, recorded as A_1_. At the same time, 5 mL of mixed tea beverage sample was placed in a 50 mL graduated colourimetric tube, 4 mL of water was added, and phosphoric acid buffer was added to a fixed volume of 25 mL. The absorbance of the solution was determined at 540 nm by ultraviolet spectrophotometer (UV-2600, Shimadzu Instrument Co., Ltd., Japan) as A_2_. The content of total phenolics in the sample was calculated according to Eq. (3).

3 $$X=\frac{({A}_{1}-{A}_{2})\times 1.957\times 2\times K}{V}\times 1000$$

Where: *X*: the content of total phenolics in the sample (mg/L); *A*_*1*_: absorbance of the test solution after colour development; *A*_*2*_: absorbance of the base colour of the test liquid; 1.957: when the absorbance was equal to 0.5 in 10 mm cuvette, the content of total phenolics in 1 mL test solution was equivalent to 1.957 mg; *K*: dilution ratio; *V*: Sample size (mL).

According to the method of Xu et al.^68^, 1 mL of the test solution was accurately absorbed and injected into a 25 mL volumetric bottle. 0.5 mL of phosphate buffer (pH = 8.04) and 0.5 mL of Ninhydrin solution were added, and the mixture was heated in a boiling water bath for 15 min. It was then cooled, and water was added to bring the volume to 25 mL. The solution was left at room temperature for 10 min, and a 10 mm colourimetric dish was used for measurement. Absorbance was measured at 570 nm as A, and a blank control was performed. According to the standard curve and regression equation, the concentration of free amino acids C (μg/mL) in the sample was calculated directly by using the measured light absorption value.

According to the method of Zhu et al.^69^, the absorbance value of tea beverage was determined using a 10 mm colourimetric cuvette at the wavelength of 660 nm with turbidity-free water as the blank solution.

According to the method of Wang, Kim, and Lee^47^, the L*, a* and b* of all samples were measured by a chromameter (CR-400, Keshengxing, Hangzhou, China). The colour parameters L*, a*, and b* represent lightness, redness/greenness, and yellowness/blueness, respectively. Before measurement, the instrument was calibrated using a black standard to set the zero point (0% reflectance) and a white standard to set the full scale (100% reflectance), ensuring that subsequent measurements are normalised within this range.

### Statistical analysis

Each experiment was repeated three times. The experimental data are presented as mean ± standard deviation. One-way ANOVA was carried out using SPSS 18.0 software, and the significance level was set at *p* < 0.05.

## Supplementary information

Supplementary materials.

## Data Availability

The datasets produced from the study are available from the corresponding author on reasonable request.
