# Supplementary material for: Increasing quality stability of jasmine tea beverage by encapsulation from protein-polysaccharide nanocomplexes
Source: NPJ Sci Food. 2026 Jan 8;10:55. doi: 10.1038/s41538-025-00703-5 (PMC12894944; doi:10.1038/s41538-025-00703-5)
Supplement: Supplementary file 1 — Supplementary materials. [file 41538_2025_703_MOESM1_ESM.docx]

**Increasing quality stability of jasmine tea beverage by encapsulation from protein-polysaccharide nanocomplexes**

Hujun Xie^a,c^, Han Wang^a^, Min Huang^a,c^, Zhenbang Zhou^a,c^, Ying Gao^b^, Qing-Qing Cao^b^, Qingbo Jiao^a^, Gerui Ren^a,c^, Yong-Quan Xu^b,*^

*^a^ School of Food Science and Biotechnology, Zhejiang Gongshang University, Hangzhou 310018, China*

*^b^ Tea Research Institute Chinese Academy of Agricultural Sciences, National Key Laboratory for Tea Plant Germplasm Innovation and Resource Utilization, Key Laboratory of Biology, Genetics and Breeding of Special Economic Animals and Plants, Ministry of Agriculture and Rural Affairs, 9 South Meiling Road, Hangzhou 310008, China*

*^c^ Key Laboratory for Food Microbial Technology of Zhejiang Province, School of Food Science and Biotechnology, Zhejiang Gongshang University, Hangzhou 310018, China.*

*Corresponding author: yqx33@126.com (Y.Q. Xu). Fax: +86−571−86650056.

**Figure S1**

**Figure S1.** Stability evaluation parameters of tea beverage under different storage conditions. (A) Total phenolic concentration, (B) Free amino acid concentration, and (C) Turbidity after storage at 25 ℃ for 30 d; (D) Total phenolic concentration, (E) Free amino acid concentration, and (F) Turbidity after storage at 4 ℃ for 150 d.

**Figure S2**

**Figure S2.** Stability evaluation parameters of tea beverage under different storage conditions. (A) *L** of colour, (B) *a** of colour, and (C) *b** of colour after storage at 25 ℃ for 30 d; (D) *L** of colour, (E) *a** of colour, and (F) *b** of colour after storage at 4 ℃ for 150 d.
